# Episodic ataxia type 2 with a novel missense variant (Leu602Arg) in *CACNA1A*

**DOI:** 10.1038/s41439-023-00261-w

**Published:** 2024-01-15

**Authors:** Shiroh Miura, Emina Watanabe, Kensuke Senzaki, Shigeyoshi Hiruki, Sayaka Matsumoto, Takuya Morikawa, Yusuke Uchiyama, Seiji Kurata, Masayuki Ochi, Yasumasa Ohyagi, Hiroki Shibata

**Affiliations:** 1 Department of Neurology and Geriatric Medicine, Ehime University Graduate School of Medicine, Shitsukawa, Toon, Ehime Japan; 2 Division of Genomics, Medical Institute of Bioregulation, Kyushu University, 3-1-1, Maidashi, Higashi-ku, Fukuoka, Japan; 3 Department of Radiology, Kurume University School of Medicine, 67 Asahi-machi, Kurume, Fukuoka, Japan

**Keywords:** Disease genetics, Spinocerebellar ataxia

## Abstract

Autosomal dominant episodic ataxia type 2 (EA2) is caused by variants in *CACNA1A*. We examined a 20-year-old male with EA symptoms from a Japanese family with hereditary EA. Cerebellar atrophy was not evident, but single photon emission computed tomography showed cerebellar hypoperfusion. We identified a novel nonsynonymous variant in *CACNA1A*, NM_001127222.2:c.1805T>G (p.Leu602Arg), which is predicted to be functionally deleterious; therefore, this variant is likely responsible for EA2 in this pedigree.

Hereditary episodic ataxia (EA) is a heterogeneous group of movement disorders characterized by recurrent spells of truncal ataxia and incoordination^1^. EA type 2 (EA2, MIM: 108500) is an autosomal dominant hereditary EA caused by heterozygous variants in the calcium voltage-gated channel subunit alpha-1A gene (*CACNA1A*, MIM: 601011). Here, we studied a patient with hereditary EA originating from Shikoku Island, Japan (Fig. 1A). The proband (Patient III-1) was a 20-year-old male who had experienced brief episodes (<30 min) of ataxia since childhood that were precipitated by actions such as running or riding a bicycle. The frequency of the episodes was once a week at most. Witnesses said that his eyes were bloodshot while he was experiencing EA. Interictal neurological examination showed no abnormalities. Brain magnetic resonance imaging (MRI) did not show obvious cerebellar atrophy (Fig. 1B). Interictal brain single photon emission computed tomography (SPECT) using N-isopropyl-p-(iodine-123)-iodoamphetamine (^123^I-IMP) with three-dimensional stereotactic surface projections showed hypoperfusion in the cerebellum, brainstem, and lateral occipital lobe (Fig. 1C). His symptoms were initially improved with acetazolamide (125 mg/day), but the effect did not last. According to the proband, his mother (II-3) and maternal grandfather (I-1) experienced the same ataxic symptoms until they were 20 years old. His 16-year-old younger brother (III-2) and 9-year-old sister (III-3) also exhibit the same ataxic symptoms.

**Fig. 1:**
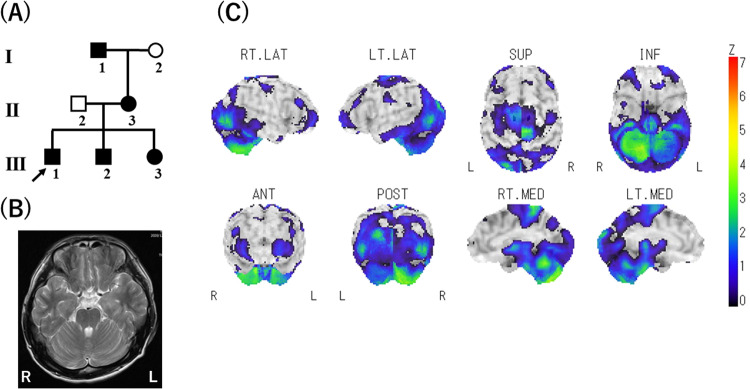
Pedigree and neuroimaging. **A** Pedigree of the EA2 family. Squares: males, circles: females, solid symbols: affected individuals, open symbols: unaffected individuals. An arrow indicates the proband. **B** Brain magnetic resonance imaging (MRI) did not show obvious cerebellar atrophy. **C** Single photon emission computed tomography (SPECT) using N-isopropyl-p-(iodine-123)-iodoamphetamine (^123^I-IMP) with three-dimensional stereotactic surface projections showed hypoperfusion in the cerebellum, brainstem and lateral occipital lobe. R right, L left, RT. LAT right lateral, LT. LAT left lateral, SUP superior, INF inferior, ANT anterior, POST posterior, RT. MED right medial, LT. MED left medial, Z Z score.

We first confirmed the absence of repeat expansion in genes known to be responsible for spinocerebellar ataxia (SCA) 1–3, 6–8, 10, 12, 17, and 36 and dentatorubral-pallidoluysian atrophy. We also confirmed the absence of pathogenic variants in SCA31. We then performed exome sequencing of the proband using SureSelect Human All Exon v6 (Agilent Technologies, Santa Clara, CA, USA) on the Illumina NovaSeq 6000 platform (Illumina, Inc., San Diego, CA, USA). We achieved a sequencing depth of 168× and identified 24,655 variants in the proband. Since the proband was diagnosed with EA, we selected 21 variants located in eight genes known to be associated with EA (Supplementary Table 1). By excluding variants already registered in public databases [the 1000 Genomes Project (http://www.1000genomes.org), ExAC (http://exac.broadinstitute.org/), and gnomAD (https://gnomad.broadinstitute.org/)], we identified a novel nonsynonymous variant located in exon 14 of the calcium voltage-gated channel subunit alpha-1A gene (*CACNA1A*), NM_001127222.2:c.1805T>G (p.Leu602Arg) (Fig. 2). The variant was predicted to be “probably damaging” by PolyPhen-2 (http://genetics.bwh.harvard.edu/pph2/), “deleterious” by SIFT (https://sift.bii.a-star.edu.sg/) and “disease-causing” by Mutation Taster (https://www.mutationtaster.org) with a CADD score of 29.4. Given the patient’s symptoms, genes known to be associated with dystonia, episodic kinesigenic dyskinesia and SCAs were also examined (Supplementary Table 1). However, no pathological variants were detected in any genes associated with these three conditions.

**Fig. 2:**
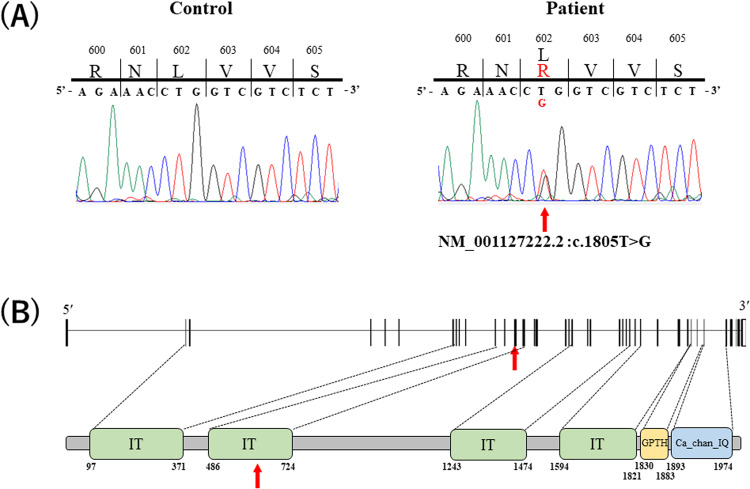
Sanger validation of the variant and the gene structure of *CACNA1A*. **A** Electropherogram of the *CACNA1A* NM_001127222.2:c.1805T>G (p.Leu602Arg) variant region in the proband (III-1) and an unaffected control. The location of the variant is indicated by a red arrow. **B** Genomic structure of the *CACNA1A* gene. The ion transport domain (IT), voltage-dependent L-type calcium channel, IQ-associated domain (GPTH) and voltage-gated calcium channel IQ domain (Ca_chan_IQ) are shown according to the NCBI Conserved Domain Search (https://www.ncbi.nlm.nih.gov/Structure/cdd/wrpsb.cgi?seqinput=NP_001120693.1). Red arrows indicate the location of the variant.

We validated the *CACNA1A* variant in the patient by Sanger sequencing (forward primer, 5′-GGGAAAGTGAGCCTCGTGT-3′ and reverse primer 5′-GGAGTTGGAATTCCTGTGAAG-3′). We confirmed that the patient was heterozygous for the variant, consistent with the autosomal dominant mode of disease inheritance (Fig. 2A). We also examined the CAG repeat length in *CACNA1A* and confirmed that the proband was homozygous for an 11-repeat allele that is nonpathogenic. According to the ACMG/AMP/CAP guidelines, the p.Leu602Arg variant is classified as “likely pathogenic”, meeting the PM1, PM2, PP3 and PP4 criteria^2^. The *CACNA1A* variant data have been deposited in ClinVar (https://www.ncbi.nlm.nih.gov/clinvar/variation/1809801/).

We described here a Japanese EA2 patient carrying a novel nonsynonymous heterozygous variant [*CACNA1A*, NM_001127222.2:c.1805T > G (p.Leu602Arg)]. The patient exhibited a typical EA2 phenotype^1,3^. The neuroradiological features of our patient included hypoperfusion of the cerebellum on brain SPECT despite no marked cerebellar atrophy on brain MRI. Brain SPECT is a functional neuroimaging technique for evaluating cerebrovascular disorders, neurodegenerative diseases, and epilepsy and may be able to detect lesions that do not produce abnormal findings on MRI^4,5^. As in the present case, brain MRI revealed no cerebellar atrophy in other EA2 cases^6,7^. To our knowledge, this is the first report of brain SPECT in a patient with EA2 caused by a heterozygous point mutation in the *CACNA1A* gene, although a brain SPECT study has been reported for a patient with familial hemiplegic migraine 1 (FHM1, MIM: 141500) carrying a heterozygous point mutation in the *CACNA1A* gene^8^. Additionally, several brain SPECT studies in patients with SCA6 (MIM: 183086) caused by a CAG trinucleotide repeat expansion in the *CACNA1A* gene have been reported^9–11^. All reported patients with *CACNA1A* variations showed atrophy and hypoperfusion localized in the cerebellum^8–11^. There are no previous reports of brain SPECT in patients with EA2; therefore, it is currently unclear whether reduced perfusion of the brainstem is common. It is important to collect brain SPECT data from more EA2 patients. The nonsynonymous variant is located within one of the ion-transport domains that is essential for the channel function of the CACNA1A protein (Fig. 2B). Although the nonsynonymous variant is predicted to be highly pathogenic by multiple prediction tools, including “probably damaging” by PolyPhen-2 and “disease-causing” by Mutation Taster, we were unable to perform segregation analysis of the variant because additional family members were not available.

We conclude that the novel *CACNA1A* variant NM_001127222.2:c.1805T>G (p.Leu602Arg) is highly likely to be responsible for EA2 in the current pedigree. Clinically, when patients complain of recurrent ataxic episodes, it is important to look for cerebellar hypoperfusion by brain SPECT even when brain MRI does not show cerebellar atrophy.

## Supplementary information

Supplementary Table 1

## Data Availability

The relevant data from this Data Report are hosted at the Human Genome Variation Database at 10.6084/m9.figshare.hgv.3357.
